# Genetic characterization of rotavirus A strains circulating in children under 5 years of age with acute gastroenteritis in Tehran, Iran, in 2023–2024: dissemination of the emerging equine-like G3P[8]-I2-E2 DS-1-like strains

**DOI:** 10.1099/jgv.0.002088

**Published:** 2025-03-18

**Authors:** Somayeh-Sadat Hosseini-Fakhr, Somayeh Jalilvand, Ali Maleki, Atefeh Kachooei, Farzane Behnezhad, Mahtab Mir-Hosseinian, Seiedehparnian Taghvaei, Sayed Mahdi Marashi, Zabihollah Shoja

**Affiliations:** 1 Department of Virology, School of Public Health, Tehran University of Medical Sciences, Tehran, Iran; 2 COVID-19 National Reference Laboratory (CNRL), Pasteur Institute of Iran, Tehran, Iran; 3 Department of Influenza and Respiratory Viruses, Pasteur Institute of Iran, Tehran, Iran; 4 Department of Virology, School of Medicine, Iran University of Medical Sciences, Tehran, Iran; 5 Department of Virology, Pasteur Institute of Iran, Tehran, Iran; 6 Pediatric Pathology Research Center, Research Institute for Children’s Health, Shahid Beheshti University of Medical Sciences, Tehran, Iran; 7 Research Center for Emerging and Reemerging Infectious Diseases, Pasteur Institute of Iran, Tehran, Iran

**Keywords:** DS-1-like, equine G3 strain, rotavirus

## Abstract

The present study was conducted to monitor the genotype diversity of circulating species A rotavirus (RVA) in Iran. A total of 300 faecal specimens were collected from children under 5 years of age hospitalized for acute gastroenteritis between October 2023 and October 2024. G3P[8] represented 72.91% (70/96) of all RVA-positive samples, further subdivided into equine-like G3P[8]-I2-E2 DS-1-like and human G3P[8]-I1-E1 Wa-like. A retrospective genetic analysis of G3P[8] strains isolated from 2015 to 2017 was also performed and showed that G3P[8] strains belong to the G3P[8]-E1-I1 Wa-like genetic pattern, which is typically similar to human G3P[8] Wa-like strains in this study. The emergence of equine-like G3P[8] DS-1-like strains in Iran may not be related to selection pressure from rotavirus vaccination, but rather to cross-border migration of rotavirus strains due to population movements.

## Data Availability

The VP7, VP4, NSP4 and VP6 nucleotide sequences of G3P[8] strains were deposited in the GenBank database under accession numbers PQ728481–PQ728606.

## Introduction

Rotavirus represents a genus of the *Sedoreoviridae* family [1]. Rotaviruses are non-enveloped, icosahedral virions consisting of 3-layered capsids enclosing 11 segments of dsRNA. Species A rotaviruses are the leading cause of acute gastroenteritis in humans and represent a major global health threat, particularly in children under 5 years of age in low–middle-income countries (LMICs) [2]. Throughout this paper, ‘rotavirus’ will be referred to as ‘species A rotavirus’. Two types of live-attenuated human oral rotavirus vaccines, Rotarix and RotaTeq, became available in 2006 [3,5]. In 2009, the World Health Organization (WHO) recommended the inclusion of rotavirus vaccines in national immunization programmes, particularly in LMICs. Gavi, the Vaccine Alliance, has subsidized the cost of rotavirus vaccines in many eligible countries [6]. In addition, two other live-attenuated human oral rotavirus vaccines, ROTAVAC and ROTASIIL, have been prequalified by the WHO and have demonstrated similar efficacy to RotaTeq and Rotarix in India and several other LMICs [7,10]. Rotavirus genotype combinations G1P[8], G2P[4], G3P[8], G4P[8], G9P[8] and G12P[8] are known to be the most common G-P genotypes causing acute gastroenteritis cases with variable geographic distribution [11,14]. The G1P[8] combination is predominant in both high-income countries and LMIC [11,12, 14].

Based on all 11 genomic RNA segment analyses, rotavirus strains are further classified into 3 genotype constellations, i.e. Wa-like (genogroup 1; G1/3/4/9/12P[8]‐I1‐R1‐C1‐M1‐A1‐N1‐T1‐E1‐H1), DS-1-like (genogroup 2; G2P[4]-I2-R2-C2-M2-A2-N2-T2-E2-H2) and AU-1-like (genogroup 3; G3P[9]-I3-R3-C3-M3-A3-N3-T3-E3-H3) [15,16], which originate from a different animal species [15]. The segmented nature of rotavirus genomes allows reassortment phenomena to occur within and between genogroups and dramatically accelerates rotavirus evolution. However, inter-genogroup reassortment occurs much less frequently than intra-genogroup reassortment among rotavirus strains [17]. It has been documented that the emergence of equine-like G3P[8] DS-1-like strains may be the result of the reassortment phenomenon. It is important to note that the majority of globally circulating G3P[8] strains had a Wa-like constellation with a G3 lineage I VP7 [15,17, 18], but there is strong support for the most likely scenario that equine-like G3P[8] DS-1-like strains originated primarily from reassortment events in the recent past with contemporary DS-1-like G1P[8] and G3P[4] DS-1-like strains as parents [19,24] and spread rapidly to several regions, including Africa, Europe, Asia, the Americas and Australia [18,23,42].

In Iran, although rotavirus vaccination was not included in the national immunization programme prior to this study, ROTASIIL oral vaccine has been included in the national immunization programme since December 2024 and is administered to all infants in three doses at 2, 4 and 6 months of age [43]. Considering the distribution of rotavirus genotypes in Iran, although G1P[8] was the most commonly detected rotavirus strain before vaccination [44], it should be noted that the major rotavirus genotypes have shifted from G1P[8] to the rare and uncommon genotypes of G3P[8] [45], G9P[8] [46] and G9P[4] [46,47] over the past 5 years, highlighting that genotype predominance is dynamic and may change over time. Consistent with our findings on stool samples collected from 2015 to 2017 [45], the G3P[8] strains were also the most prevalent genotype in this study. Since most human infections linked to equine-like G3P[8] have DS-1-like backbone genes, this study aims to characterize the genetic variants VP7 and VP4 segment and genotypic patterns of VP6 and NSP4 of detected G3P[8] strains as well as reported G3P[8] from stool samples collected from 2015 to 2017. A comparative analysis of VP4 and VP7 lineages and antigenic sites of circulating and vaccine strains was also conducted to monitor rotavirus trends.

## Methods

### Specimen collection

Between October 2023 and October 2024, a total of 300 faecal samples were collected from children under 5 years of age hospitalized for acute gastroenteritis at Tehran Children’s Hospital. Inclusion criteria for this study include all hospitalized children (0–5 years of age) diagnosed with suspected rotavirus-related acute gastroenteritis, defined as the passing of ≥3 watery or loose stools each day. All faecal samples were transported to the laboratory of the Molecular Virology Division of the Pasteur Institute of Iran and stored at −20 °C until processed for rotavirus detection and molecular analysis. The agreement and informed consent were obtained from the parents of all children under 5 years of age hospitalized for acute gastroenteritis at Tehran Children’s Hospitals. The study was conducted according to the Helsinki guidelines and approved by the ethics committee of the Pasteur Institute of Iran and Tehran University of Medical Sciences (IR.TUMS.SPH.REC.1402.218).

### Viral RNA extraction and cDNA synthesis

For viral RNA extraction, faecal specimens were first diluted as a 10% (w/v) faecal suspension in PBS (pH 7.4) and then centrifuged at 1,500 ***g*** for 20 min. The supernatants were used to extract viral RNA using the High Pure Viral RNA Kit (Roche Diagnostics GmbH, Germany) according to the manufacturer’s instructions. The extracted dsRNA was denatured by heating at 97 °C for 5 min and rapidly quenched in a dry ice-ethanol bath. The cDNA was synthesized using the cDNA Synthesis Kit (Cat No: YT4500; Yekta Tajhiz Azma Company, Tehran, Iran) in a two-step approach in a final volume of 20 µl, according to the manufacturer’s instructions.

### Rotavirus detection and genotype determination

Rotavirus screening was performed by reverse transcription polymerase chain reaction (RT-PCR) using VP6-F: 5′-GACGGNGCRACTACATGGT-3′ and VP6-R: 5′-GTCCAATTCATNCCTGGTGG-3′ with the expected size of 379 bp as described by Iturriza Gómara *et al*. [48]. Positive samples were then subjected to G and P typing by semi-nested multiplex RT-PCR with type-specific primers according to the standard method for the detection and characterization of rotavirus [49,50]. All reaction/PCR conditions for the first and semi-nested rounds were as described in our previous studies [45,51]. The expected sizes of the VP7 (first round: 1,062 bp) and the VP4 (first round: 887 bp) PCR products were analysed by agarose gel electrophoresis. Amplified products corresponding to the target segment size were used for G (second round; G : 749 bp, G2: 652, G3: 812 bp, G4: 583 bp, G8: 885 bp and G9: 306 bp) and P (second round; P[8]: 345 bp, P[4]: 483 bp, P[6]: 267 bp, P[9]: 391 bp and P[10]: 594 bp) genotyping of rotavirus strains.

For I and E genotyping, the amplification of the VP6 and NSP4 genes was performed by semi-nested RT-PCR using VP6- and NSP4-specific primers as described by Kachooei *et al*. [51]. The expected size of the VP6 (first round: 1,194 bp, second round: 1,103) and NSP4 (first round: 750 bp, second round: 566) PCR products was verified by agarose gel electrophoresis.

### Sequence analysis

To understand variants of circulating G3 strains as a dominant strain in this study, samples (*n*=47) meeting the established criteria [PCR amplification of the full-length VP7 gene (1,062 bp) and the partial VP4 gene (VP8* region; 877 bp)] were selected for Sanger sequencing of their VP7 and VP4 genes. Subsequently, VP7 sequences (i) previously published by Ghanaiee *et al*. [47] (*n*=7; samples collected in 2022), (ii) our previous study by Motamedi-Rad *et al*. [45] (*n*=16; samples collected from 2015 to 2017), (iii) newly obtained in this study (*n*=47; samples collected from October 2023 to October 2024) and (iv) G3 lineages I–IX as reference sequences extracted from GenBank [52,53] were combined, and the query sequences in this dataset were genotyped in a phylogenetic analysis based on their clustering with the reference sequences in the maximum likelihood method (MLM). Meanwhile, the VP4 gene of the G3P[8] strains in this study was compared with human rotavirus VP4 sequences of P[8] previously known from four lineages (I–IV) [54] and also genotyped in a phylogenetic analysis.

In addition, to identify G3 strains with possible Wa-like or DS-1-like patterns, VP6 and NSP4 gene segments from samples genotyped as G3P[8] were selected for Sanger sequencing. The nt sequence of the amplified products VP7, VP4, VP6 and NSP4 was determined by sequencing with the BigDye Terminator v3.1 Cycle Sequencing Kit on a 3130 Genetic Analyzer Automated Sequencer according to Applied Biosystems protocols (Applied Biosystems, Foster City, CA, USA) and edited with CLC Main Workbench (CLC Bio, v5.5). Multiple sequence alignment was performed using reference sequences from GenBank, and phylogenetic analysis was performed using the MLM based on the Kimura two-parameter model (mega 11) [55]. The tree was measured using the bootstrap method with 1,000 replicates. The accession numbers of VP7, VP4, VP6 and NSP4 sequences were deposited in GenBank (accession numbers PQ728481–PQ728527 for VP7, accession numbers PQ728528–PQ728574 for VP4, accession numbers PQ728575–PQ728590 for NSP4 and accession numbers PQ728591–PQ728606 for VP6).

## Results

### Distribution of circulating G-P genotypes

Of the 300 faecal samples analysed, 96 (32%) tested positive for rotavirus. G genotyping by RT-PCR identified G3 as the most prevalent genotype (76/96; 79.2%), followed by G1 (6/96; 6.2%), G9 (2/96; 2.08%), G8 (1/96; 1.04%), G12 (2/96; 2.08%) and untyped strains (Gx; 9/96; 9.4%) (Table 1). Using RT-PCR for P genotyping, ~86.43% (83/96) of these positive samples were assigned to the P[8] genotype, followed by P[6] (1/96; 1.04%), P[9] (1/96; 1.04%) and P[x] (untyped, 11/96; 11.5%). A total of six G and P genotype combinations were identified, including G3P[8], G1P[8], G3P[9], G8P[8], G9P[8] and G12P[6]. Among them, G3P[8] (70/96; 72.91%) was the most frequently detected genotype combination (Table 1).

**Table 1.** Distribution of rotavirus genotypes in hospitalized children under 5 years of age from October 2023 to October 2024, *n* (%)

P type	G type						
	G1	G3	G8	G9	G12	Gx*	Total
P[8]	5 (5.2)	70 (72.91)	1 (1.04)	1 (1.04)	1 (1.04)	5 (5.2)	83 (86.43)
P[6]	0 (0)	0 (0)	0 (0)	0 (0)	1 (1.04)	0 (0)	1 (1.04)
P[9]	0 (0)	1 (1.04)	0 (0)	0 (0)	0 (0)	0 (0)	1 (1.04)
P[x]*	1 (1.04)	5 (5.2)	0 (0)	1 (1.04)	0 (0)	4 (4.2)	11 (11.5)
Total	6 (6.2)	76 (79.2)	1 (1.04)	2 (2.08)	2 (2.08)	9 (9.4)	96 (100)

*Values in parenthesis indicate the percentage of the total of 96.

* Gx and P[x] indicate untyped.

### The VP7 and VP4 sequence analyses of G3P[8] strains

**Fig. 1.**
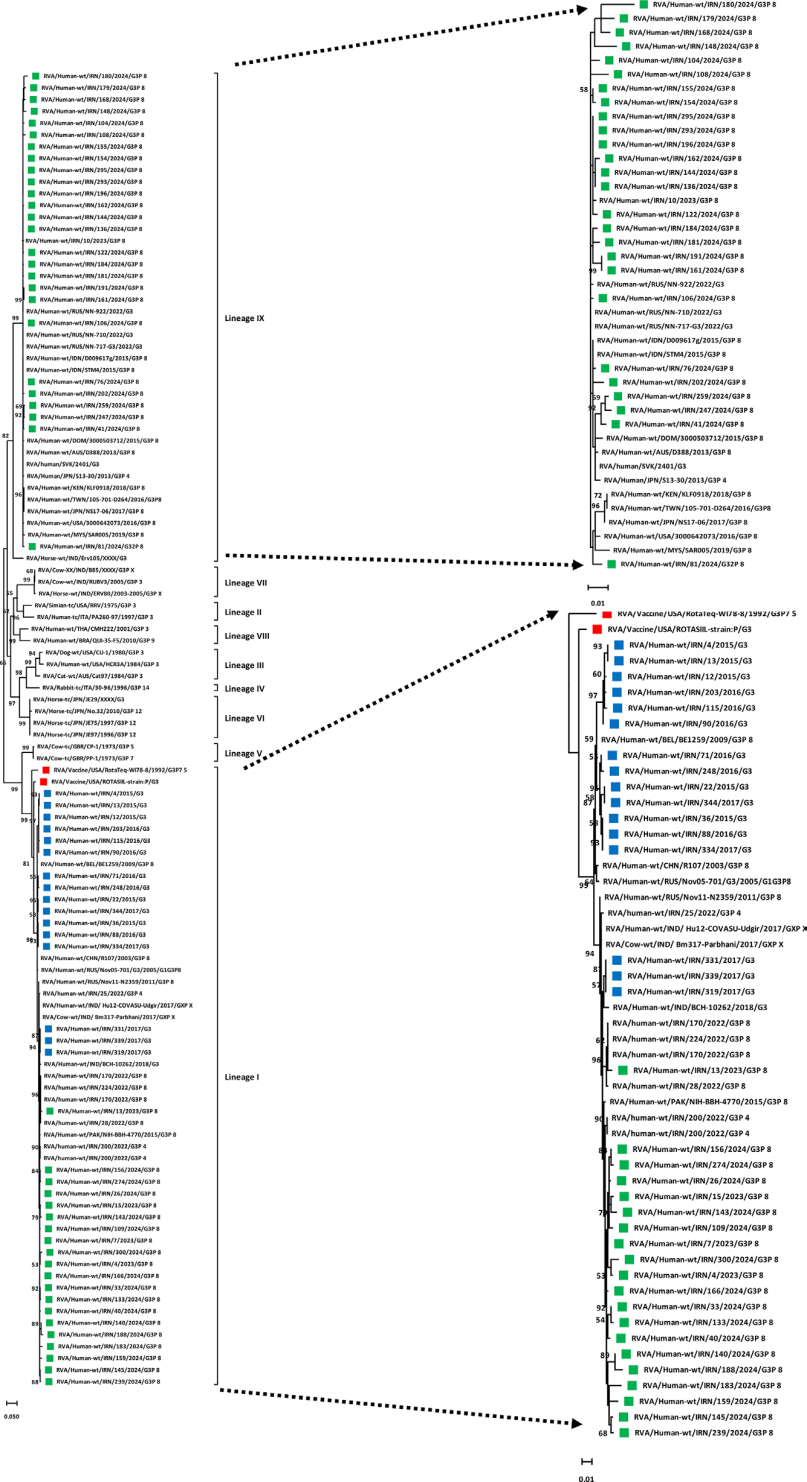
Phylogenetic analysis based on the nt sequences of the VP7 gene of detected G3P[8] strains in Iran. Phylogenetic tree analyses for VP7 nt sequences were inferred by using the MLM with 1,000 bootstrap replicates. Significant bootstrap values are indicated. The scale bar represents 5% genetic distance. The sequences of rotavirus strains identified in this study (October 2023–October 2024), previous studies (2015–2017 and 2022) and vaccine strains (RotaTeq and ROTASIIL) are indicated by their strain names with green, blue and red squares, respectively. The reference strains are indicated by the strain names.

**Fig. 2.**
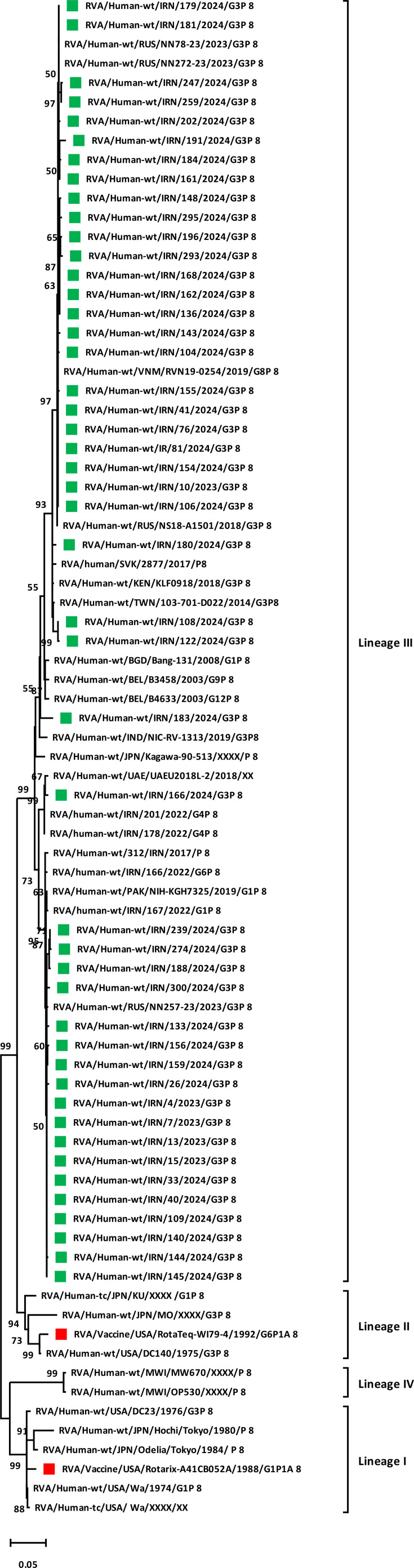
Phylogenetic analysis based on the nt sequences of VP4 (VP8*) gene of detected G3P[8] strains in Iran. Phylogenetic tree analyses for VP4 (VP8*) nt sequences were inferred by using the MLM with 1,000 bootstrap replicates. Significant bootstrap values are indicated. The scale bar represents 5% genetic distance. The sequences of rotavirus strains identified in this study (October 2023–October 2024) and vaccine strains (Rotarix and RotaTeq) are indicated by their strain names with green and red squares, respectively. The reference strains are indicated by the strain names.

The phylogenetic tree of VP7 was constructed and showed that the Iranian G3 strains in the present study (October 2023–October 2024) clustered into two different lineages, human-like G3 lineage I (*n*=20) and equine-like G3 lineage IX (*n*=27), statistically supported by 99% and 82% bootstrap values (Fig. 1). In particular, the Iranian equine-like G3 strains clustered with the Indian equine-like G3 strains (RVA/Horse-wt/IND/Erv105/XXXX/G3) and formed polyphyletic subclusters with Australian, Dominican Republic, Indian, Japanese, Kenyan, Russian, Taiwanese and US equine-like G3 strains with the highest nt/aa homology [with average nt identity 99.09% (96.9–99.8%) and average aa identity 99.17% (97.2–100)]. The Iranian human-like G3 lineage I also clustered with sequences from Russia, India and Pakistan with the highest nt/aa homology [with average nt identity 97.5% (92.6–99.5%) and average aa identity 97% (91.2–99.6)]. Moreover, the phylogenetic analysis of the VP4 gene of the Iranian G3P[8] strains showed that they clustered within lineage III and were closely related to strains from Iran, Russia, Slovakia, Kenya, Vietnam, Taiwan and Pakistan with the highest nt homology [with average nt identity of 96.8% (93.5–100%) and average aa identity of 97.6% (94.4–100%)], statistically supported by 99% bootstrap values (Fig. 2).

### The VP6 and NSP4 sequence analyses of G3P[8] strains

**Fig. 3.**
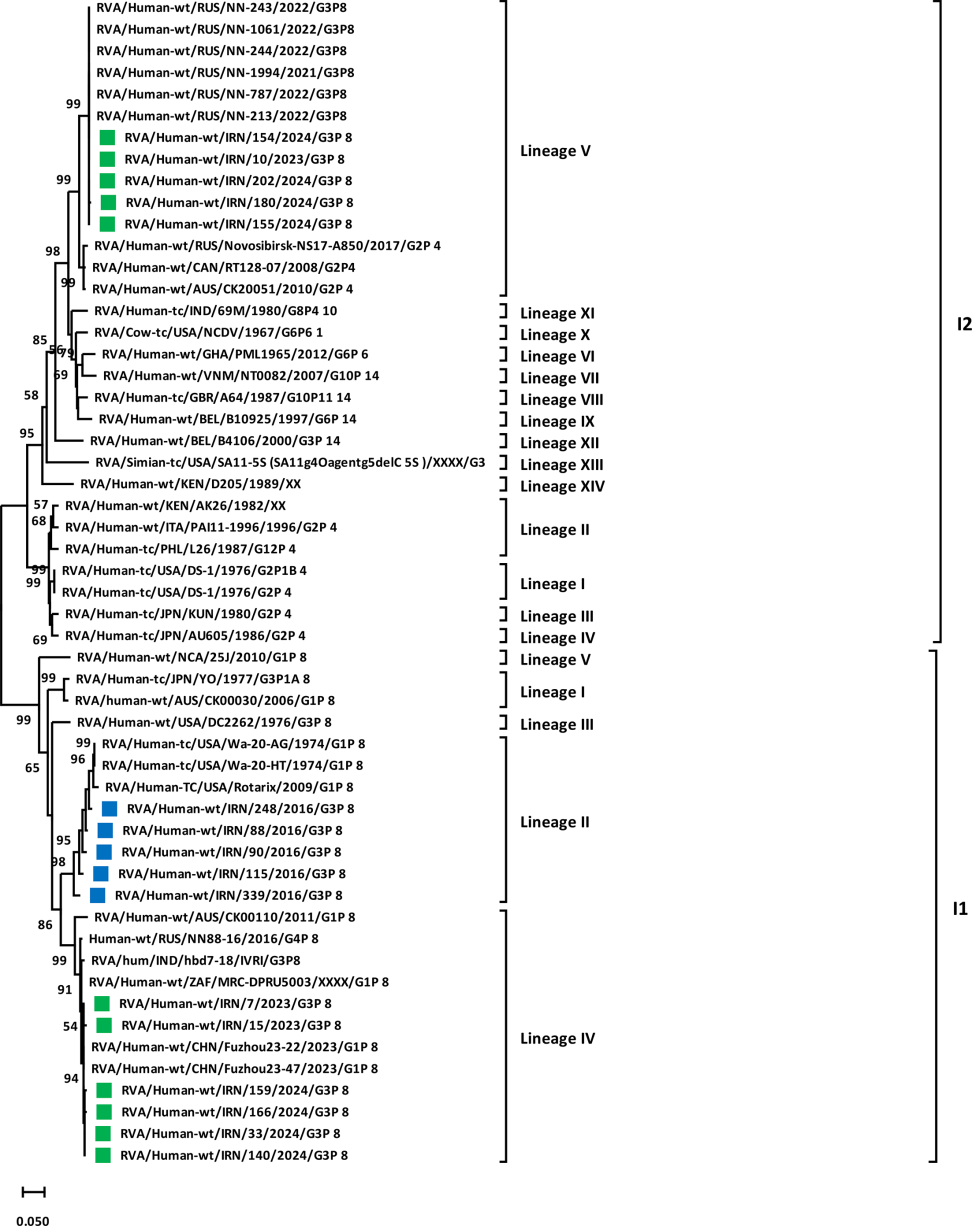
Phylogenetic analysis based on the nt sequences of the VP6 gene of detected G3P[8] strains in Iran. Phylogenetic tree analyses for VP6 nt sequences were inferred by using the MLM with 1,000 bootstrap replicates. Significant bootstrap values are indicated. The scale bar represents 5% genetic distance. The sequences of rotavirus strains identified in this study (October 2023–October 2024) and previous studies (2015–2017 and 2022) are indicated by their strain names with green and blue squares, respectively. The reference strains are indicated by the strain names.

To better understand the genetic evolution and possible genogroup pattern (Wa-like or DS-1-like) of G3 strains circulating in Iran, VP6 and NSP4 analyses of G3P[8] strains were also performed (Table 2). In this regard, VP6 and NSP4 sequences of 11 G3 strains detected from October 2023 to October 2024 (6 human-like G3P[8] and 5 equine-like G3P[8] strains) and 5 G3 strains (human-like G3P[8]) detected from 2015 to 2017 were determined and compared with closely related VP6 and NSP4 sequences in the GenBank database. Phylogenetic analysis of VP6 showed that equine-like G3P[8] strains belong to the I2 genotype cluster, which was statistically supported by a 99% bootstrap value (Fig. 3). It was also confirmed that within the I2 lineage V cluster, VP6 from equine strains formed a sub-cluster together with typical DS-1-like strains, including RVA/Human-wt/RUS/NN-243/2022/G3P[8], RVA/Human-wt/RUS/NN-1061/2022/G3P[8], RVA/Human-wt/RUS/NN-244/2022/G3P[8], RVA/Human-wt/RUS/NN-1994/2021/G3P[8], RVA/Human-wt/RUS/NN-787/2022/G3P[8] and RVA/Human-wt/RUS/NN-213/2022/G3P[8] from Russia with highest nt homology. Furthermore, this analysis documented that all human-like G3P[8] strains fell into the I1 genotype cluster, which was statistically supported by 99% bootstrap values (Fig. 3). Within cluster I1, human-like G3P[8] strains detected in 2015–2017 formed a polyphyletic subcluster together with typical Wa-like and Rotarix strains in G3 lineage II, which was statistically supported by 98% bootstrap value, while human-like G3P[8] strains detected in 2023–2024 formed a subcluster in G3 lineage IV, statistically supported by 99% bootstrap value, and were closely related to sequences from China (RVA/Human-wt/CHN/Fuzhou23-22/2023/G1P[8] and RVA/Human-wt/CHN/Fuzhou23-47/2023/G1P[8]) (Fig. 3).

**Table 2.** Characterization of circulating variants G3 in Iran

	Strain	VP7	VP4	VP6	NSP4	Wa-like/DS-1-like
**Present study**	RVA/Human-wt/IRN/*n*=27/2023–2024/G3P[8]	Equine-like G3 lineage IX	P[8] lineage III	I2	E2	DS-1-like
	RVA/Human-wt/IRN/*n*=20/2023–2024/G3P[8]	Human typical G3 lineage I	P[8] lineage III	I1	E1	Wa-like
**Our previous study**	RVA/Human-wt/IRN/*n*=16/2015–2017/G3	Human typical G3 lineage I	P[8] lineage III	I1	E1	Wa-like
**Ghanaiee *et al.* study**	RVA/human-wt/IRN/*n*=7/2022 /G3	Human typical G3 lineage I	P[8]/P[4]	nd	nd	–

nd, not determined.

**Fig. 4.**
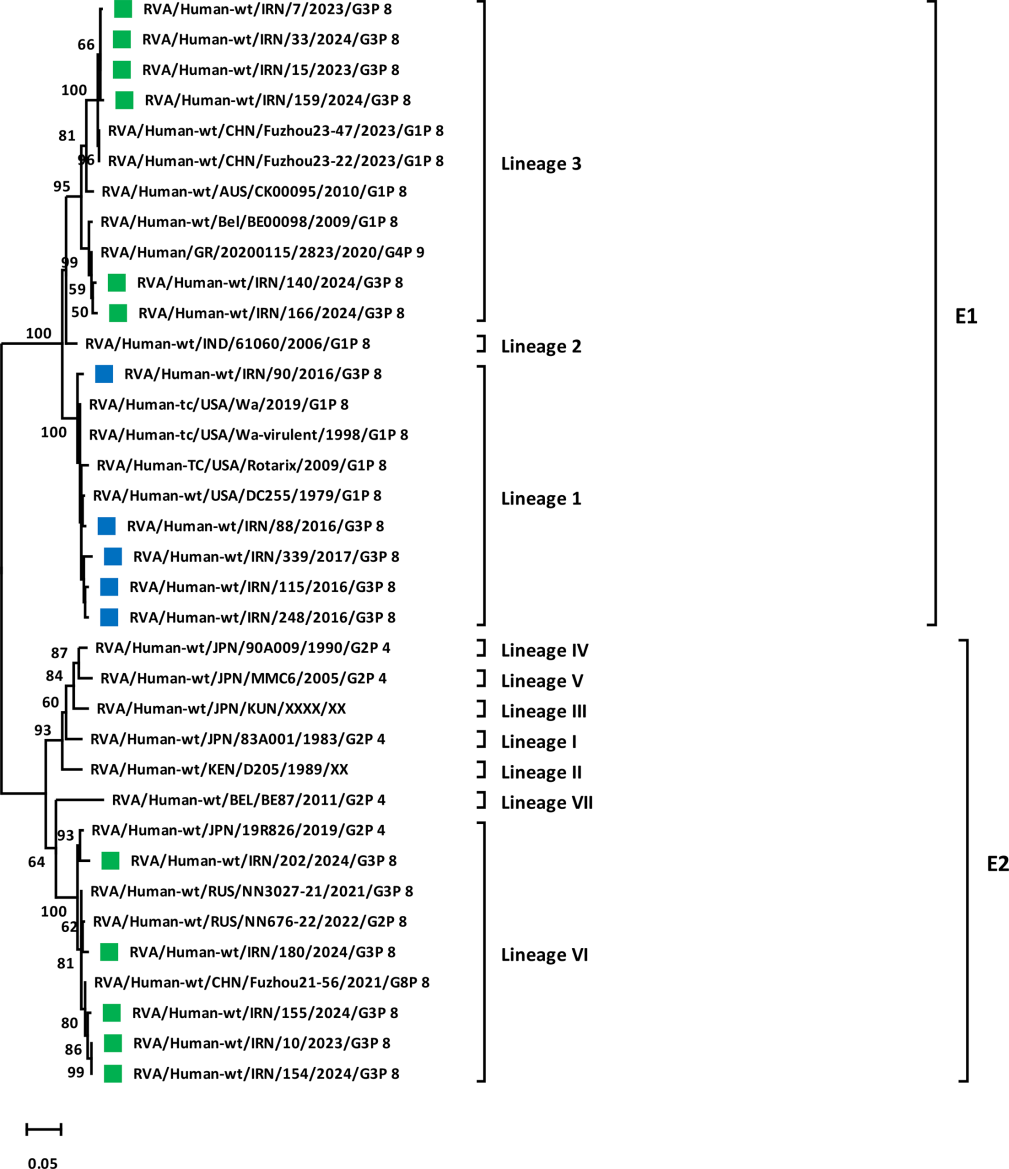
Phylogenetic analysis based on the nt sequences of the NSP4 gene of detected G3P[8] strains in Iran. Phylogenetic tree analyses for NSP4 nt sequences were inferred by using the MLM with 1,000 bootstrap replicates. Significant bootstrap values are indicated. The scale bar represents 5% genetic distance. The sequences of rotavirus strains identified in this study (October 2023–October 2024) and previous studies (2015–2017 and 2022) are indicated by their strain names with green and blue squares, respectively. The reference strains are indicated by the strain names.

The phylogenetic trees for NSP4 showed that equine-like G3P[8] strains belonged to the E2 lineage VI cluster, which was statistically supported by a 100% bootstrap value (Fig. 4). Moreover, it was confirmed that within the E2 lineage VI cluster, Iranian strains formed polyphyletic subclusters together with typical DS-1-like strains, including RVA/Human-wt/RUS/NN3027-21/2021/G3P[8], RVA/Human-wt/RUS/NN676-22/2022/G2P[8], RVA/Human-wt/CHN/Fuzhou21-56/2021/G8P[8] and RVA/Human-wt/JPN/19R826/2019/G2P[4] from Russia, China and Japan with the highest nt homology. Furthermore, NSP4 of human-like G3P[8] strains was shown to belong to the E1 genotype branch, which was statistically supported by a 100% bootstrap value (Fig. 4). Within the E1 genotype branch, human-like G3P[8] strains detected in 2015–2017 belonged to E1 lineage 1, forming a sub-cluster with typical Wa-like strains, which was statistically supported by a 100% bootstrap value, while human-like G3P[8] strains detected in this study (2023–2024) are sub-clustered in E1 lineage 3 together with human strains, including RVA/Human-wt/CHN/Fuzhou23-47/2023/G1P[8], RVA/Human-wt/CHN/Fuzhou23-22/2023/G1P[8] and RVA/Human/GR/20200115/2823/2020/G4P[9] from China and Greece with the highest nt homology.

### Comparative analysis of VP7 and VP4 antigenic epitopes of G3P[8] strains between Iran and vaccine strains

aa substitutions in the rotavirus VP7 and VP4 antigenic sites may affect the neutralizing capacity of antibodies against VP7 and VP4 and may also undermine vaccine efficacy. For the VP7 glycoprotein, two antigenic domains 7-1 and 7-2 of 29 aa have been documented, and the 7-1 domain is further subdivided into 7-1a and 7-1b [56]. The VP4 spike is proteolytically cleaved into two fragments, VP8* and VP5*. VP8*, the globular head of the spike, contains four antigenic domains (8-1, 8-2, 8-3 and 8-4) of 25 aa [57]. Of the 29 aa residues of the VP7 antigenic epitopes of the G3 strains, 6 acid residues (87, 212, 213, 238, 242 and 221) differed between all Iranian and vaccine strains (Table 3). Analysis of the Iranian human-like G3 strains (lineage I) revealed four aa substitutions of A212T, K238N, D242N and A221D compared with RotaTeq G3 and 1 substitution of A221D compared with ROTASIIL G3, all located in the 7-1b and 7-2 epitopes. Analysis of Iranian equine-like G3 (lineage IX) and RotaTeq vaccine strains showed that equine-like G3 strains only had one substitution (T87S) in the 7-1a region. Three substitutions (N213T, K238D and D242A) were detected within the 7-1b epitope of equine-like G3 strains. Additionally, compared with ROTASILL G3 strains, Iranian equine-like G3 strains showed that the antigenic epitopes of VP7 were limited by one aa substitution (T87S) in 7-1a and four aa substitutions (T212A, N213T, N238D and N242A) in 7-1b antigenic sites. Examination of the VP8* epitopes of P[8] strains showed that they contained up to nine (S146S/G, N150D, N194N/D, N195G, R183G, N113D, S125N, S131R and N135D) aa differences with Rotarix and six differences (S146S/G, N150D, N194N/D, D195G, R183G and N113D) with RotaTeq, distributed among the three antigenic epitope sites (Table 4).

**Table 3.** Alignment of the deduced aa of antigenic sites in VP7 between the G3P[8] strains contained in RotaTeq/ROTASIIL and circulating in Iran

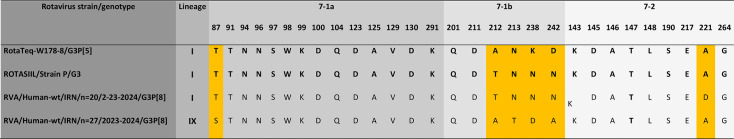 ­

Amino acid residues highlighted in yellow differ from RotaTeq and/or ROTASIIL.

**Table 4.** Alignment of the deduced aa of antigenic sites in the fragment VP8* of the VP4 gene between the G3P[8] strains contained in Rotarix and RotaTeq and circulating in Iran

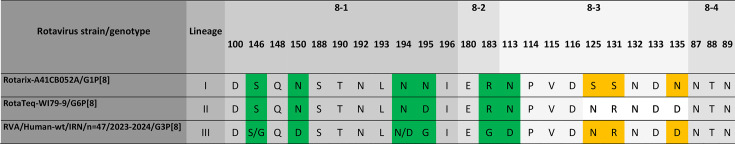 ­

Amino acid residues highlighted in green/yellow differ from Rotarix and/or RotaTeq.

## Discussion

In this study, G3P[8] strains were found to be the most common G/P combination. Although G9P[8] and G9P[4] were the most common genotypes in Iran in 2017–2023 [47,51], the dominance of G3P[8] in this study (October 2023–October 2024) was consistent with our previous data in 2015–2017 [45]. Several countries have reported an increase in the frequency of G3 strains after rotavirus vaccination (especially Rotarix) [34,36, 40, 58,60], and the highest occurrence of these novel G3 strains occurred in populations with high vaccine coverage [23]. This raises the question of whether vaccines have exerted selective pressure on these strains. However, in Iran, the emergence of these novel strains appears to be due to the natural fluctuation of rotavirus strains and is not related to the impact of rotavirus vaccine pressure. In Iran, G1P[8] combinations were continuously detected from 2001 to 2023, with a decreasing trend from 2012 to 2023 [44]. It seems that a strong homotypic immunity against G1P[8] has been induced in children, and a lower susceptibility to a second infection with a G1 strain compared to an infection with a heterotypic strain (G3P[8]) is expected. Our results suggest that the dominance of G3P[8] may fluctuate during the period 2015–2024. In support of this annual fluctuation, several previous studies have shown that the dominant strains of rotavirus naturally fluctuate over a period of 3–11 years [61,64].

The Iranian G3P[8] strains were further differentiated into DS-1-like and Wa-like patterns according to confirmatory G3 VP6 and NSP4 sequencing and genetic analysis. In particular, the detected novel equine-like G3P[8] DS-1-like strains (G3 lineage IX-P[8] lineage III-I2 lineage V-E2 lineage VI) showed a close genetic relationship with previously identified contemporary equine-like G3 strains from Russia, India and Japan, suggesting the global spread of the combination of P[8] and equine-like G3. It should be noted that the Iranian equine-like G3P[8] DS-1 strains had the same genetic makeup and were closely related to Russian equine-like G3 rotavirus strains. Alternatively, it is possible that these equine-like G3 strains were imported directly from abroad and may be due to cross-border migration of rotavirus strains due to population movements. To identify the possible entry of these equine-like G3 strains into Iran, a retrospective genetic analysis of G3P[8] strains isolated from 2015 to 2017 was performed and showed that G3P[8] strains belong to the Wa-like genetic backbone (G3 lineage I-P[8] lineage III-I1-E1 l), which is typically similar to human G3P[8] Wa-like strains in this study (October 2023–October 2024) and several other studies [15,17, 65]. Although G3 strains (*n*=2) [66] were first detected in Iran from 2006 to 2007 samples, our genetic data from 2015 to 2024 on existing G3 strains show that equine-like G3 strains emerged no later than October 2023. Therefore, it could be speculated that the spread of equine strains in Iran started in 2023. The gradual change from human G3P[8] Wa-like strains to equine-like G3P[8] DS-1-like strains from October 2023 to October 2024 may reveal a changing pattern of circulating strains, indicating the emergence of novel equine-like G3P[8] DS-1-like strains in Iran, which will continue to predominate in the coming seasons. It is worth mentioning that as an important limitation of the Iranian G3 strains in this study, it was not possible to perform a complete genome sequence analysis, and some information about the characterization of other segments in G3 rotavirus strains may have been lost.

In this study, the antigenic profiles of G3P[8] strains were characterized for VP7 and VP4 (VP8*) antigenic epitopes. In this context, aa substitutions in the 7-1a (T87S) and 7-1b (N213T, K238D and D242A) regions were observed in the VP7 antigenic epitopes of Iranian equine-like G3 strains compared to the RotaTeq G3 strain, which appear to be conserved among most equine-like G3 strains [23,26]. Compared with ROTASIIL, in addition to the four substitutions mentioned above (T87S, N213T, N238D and N242A), one residue was mutated in the 7-1b region (T212A). Furthermore, the comparison of the aa sequences of the Iranian human-like G3 strain and the G3 component of RotaTeq revealed three and one aa substitutions located in the 7-1b (A212T, K238N and D242N) and 7-2 (A221D) regions, respectively, whereas the Iranian human-like G3 rotavirus strain with only one substitution A221D in the 7-2 region was closely related to the G3 strain of ROTASIIL. The K238N aa substitution represents a possible N-linked glycosylation site, similar to previous studies [45,57, 67, 68], which influences neutralizing activity against homotypic rotavirus strains [69,70]. The comparison of the VP8* epitope sites of the Iranian P[8] strain with P[8] from Rotarix (lineage I) or RotaTeq (lineage II) revealed a significant number of differences at known neutralization escape mutation sites (S146S/G, N150D, N194N/D, N195G, R183G, N113D, S125N, S131R and N135D), which have also been reported previously [40]. Given this pattern in the deduced aa of the VP8 antigenic sites, the Iranian P[8] strains were more closely related to RotaTeq P[8] (six substitutions) than to Rotarix vaccine P[8] (nine substitutions). In the context of vaccination in Iran, where the ROTASIIL vaccine was chosen for vaccination, human G3 lineage I strains identified in Iran are closely related to the G3 strains of ROTASIIL, which belong to the same lineage (with only one substitution in the VP7 antigenic site). However, there are five aa substitutions in the equine-like G3 strains of different lineage (lineage IX) identified in Iran when compared to ROTASIIL, which may be important for the virus to escape host immunity.

Overall, to the best of our knowledge, this is the first report on the spread of the novel equine-like G3P[8] containing DS-1-like (genogroup 2) based on the I2 and E2 genetic pattern in Iran, which provides direct evidence of the switch from human G3P[8] Wa-like strains to equine-like G3P[8] DS-1-like strains during October 2023 to October 2024. Although the emergence of equine-like G3P[8] DS-1-like strains in Iran may not be related to selection pressure from the rotavirus vaccination, a steady increase in the dominance of these equine-like G3 strains in the future as an epidemiologically important strain also raises concerns about the efficacy of the rotavirus vaccine and the optimal rotavirus vaccination policy.

## Abbreviations

LMIC: low–middle-income country
MLM: maximum likelihood method
RT-PCR: reverse transcription polymerase chain reaction
RVA: species A rotavirus
WHO: World Health Organization
